# Nickel-metalated porous organic polymer for Suzuki–Miyaura cross-coupling reaction†

**DOI:** 10.1039/c9ra03679b

**Published:** 2019-06-28

**Authors:** Ying Dong, Jing-Jing Jv, Yue Li, Wen-Han Li, Yun-Qi Chen, Qian Sun, Jian-Ping Ma, Yu-Bin Dong

**Affiliations:** College of Chemistry, Chemical Engineering and Materials Science, Collaborative Innovation Centre of Functionalized Probes for Chemical Imaging in Universities of Shandong, Key Laboratory of Molecular and Nano Probes, Ministry of Education, Shandong Normal University Jinan 250014 P. R. China dongyinggreat@163.com yubindong@sdnu.edu.cn

## Abstract

A new Ni(ii)-α-diimine-based porous organic polymer, namely Ni(ii)-α-diimine-POP, was constructed in high yield *via* the Sonogashira coupling reaction between the metallo-building block of Ni(ii)-α-diimine and 1,3,5-triethynylbenzene. Besides the high thermal and chemical stability, the obtained Ni(ii)-α-diimine-POP can be a highly active reusable heterogeneous catalyst to promote the Suzuki–Miyaura coupling reaction. The obtained results indicate that the Ni(ii)-α-diimine-POP herein is a promising sustainable alternative to the Pd-based catalysts for catalysing the C–C formation in a heterogeneous way.

## Abstract 2

A porous organic polymer with Ni(ii) α-diimine moiety, which can be a highly active reusable heterogeneous catalyst to promote the Suzuki–Miyaura coupling reaction, was reported.

## Graphical abstract

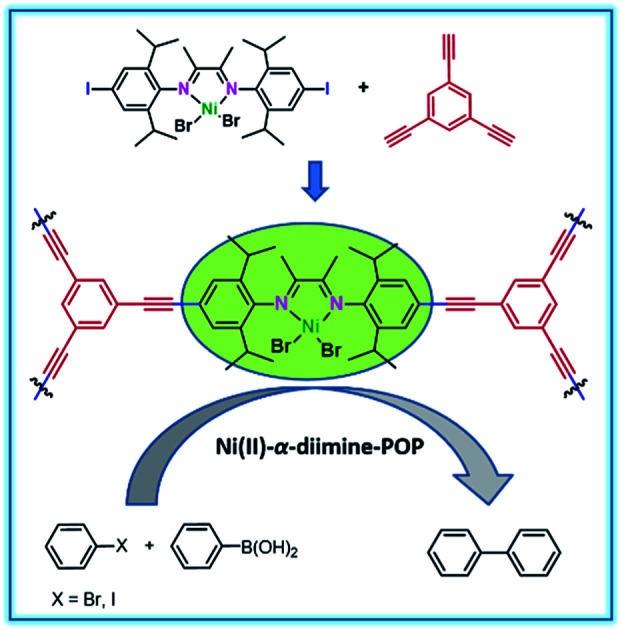

## Introduction

Homogeneous metal catalysts play a leading role in C–C bond-forming reactions.^1*a*^ Due to the increasing environmental issues and need for sustainable development, the metal-involving heterogeneous catalysts for C–C coupling such as in the Suzuki–Miyaura cross coupling reaction have drawn more and more attention during the past several decades. Among them, the precious palladium-based catalysts have been the protagonist.^1*b*^ To date, various solid carriers such as metal–organic frameworks (MOFs), covalent organic frameworks (COFs), carbon-based nanomaterials (CNMs), silica, and so on were employed to support precious Pd complex,^2^ Pd NP^3^ and Pd-bimetallic alloy^4^ for the fabrication of Pd heterogeneous catalysts. In contrast, the first row transition metals such as Ni are rarely used to fabricate the solid carrier-supported heterogeneous catalysts. Compared to Pd, Ni is more reactive, low-cost and earth-abundant. As a sustainable alternative to Pd, the Ni-based catalysts, especially solid carrier supported Ni metal catalysts, for the C–C cross-coupling reactions are extremely appealing.^5^

The α-diimine-based metal catalysts have gained remarkable attention because of their easy synthesis, air stability, and high catalytic activity.^6^ For example, α-diimine Ni(ii) complexes have been widely used in polymerization,^7^ dry CO_2_ reforming of methane,^8^ chemical bond activation,^9^ bimetallic catalysis,^10^ reductive cross-coupling reaction,^11^ and reductive amination.^12^

On the other hand, porous organic polymers (POPs), as a typical class of porous organic materials, have been broadly applied in the field of gas storage and separation,^13^ drug delivery,^14^ water treatment^15^ and heterogeneous catalysis.^16^ On account of their high surface area, controllable porosity and ability to be functionalized, both crystalline and amorphous POPs, such as covalent organic frameworks (COFs),^17^ hypercrosslinked polymers (HCP),^18^ porous aromatic frameworks (PAFs),^19^ polymers of intrinsic microporosity (PIMs),^20^ conjugated microporous polymers (CMP),^21^ are an promising class of carriers to upload active catalytic species. So far, the metal nanoparticle (M NP) loaded and metalated POPs by post-synthetic approach have been the main theme in the fabrication of POP-supported metal-solid catalysts.^22^ In principle, the POP-based and metal-involved catalytic materials could also be prepared by *in situ* one-pot assembly of metal-containing building blocks. By doing so, the POPs with high-density and evenly distributed metal catalytic sites would be generated. So far, the metalated POPs obtained in this way, however, are very rarely reported.^23^

In this contribution, we report, the first of its kind, a Ni(ii)-POP which was generated from the metallo-building block of Ni(ii) α-diimine and 1,3,5-triethynylbenzene *via* Sonogashira cross-coupling reaction under solvothermal conditions. The obtained Ni(ii)-α-diimine-POP can be a highly active and reusable heterogeneous catalyst to promote Suzuki–Miyaura cross-coupling reactions.

## Results and discussion

### Structural and morphological characterization

**Scheme 1:**
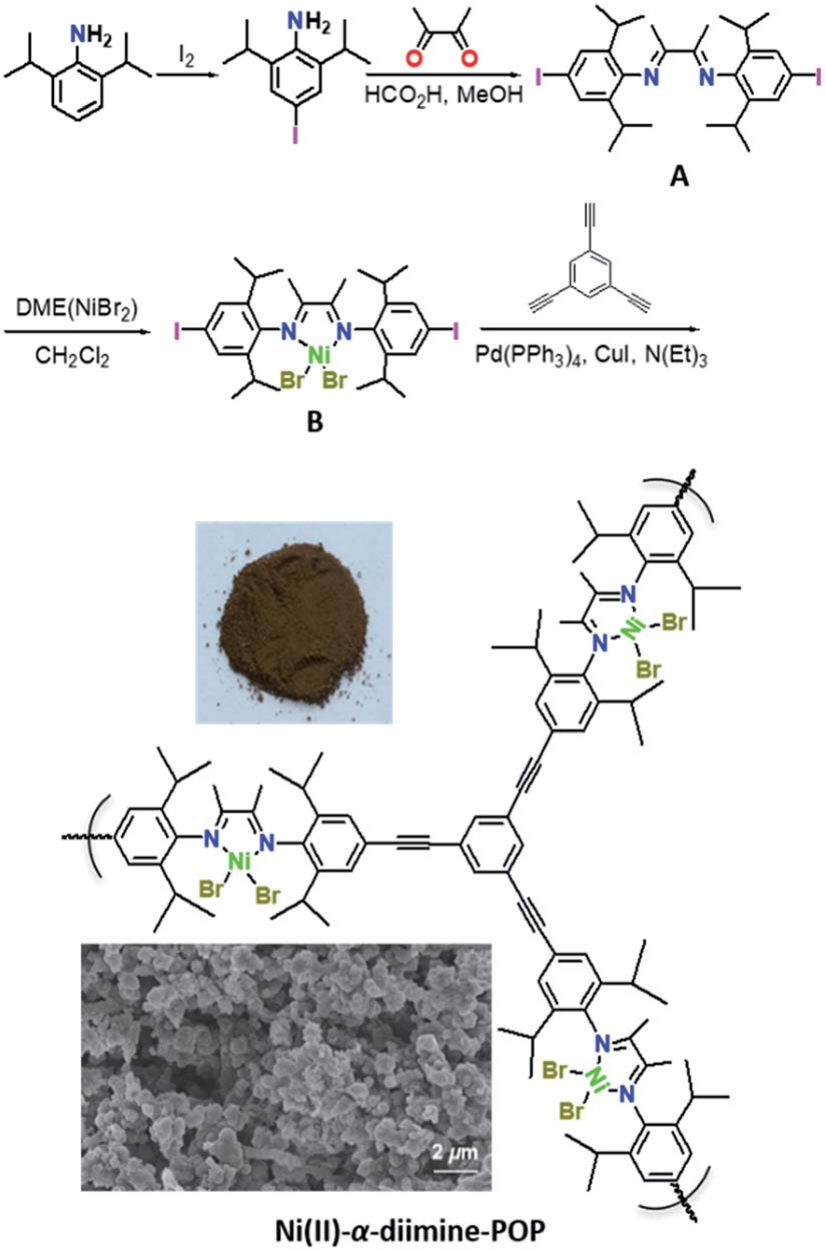
Synthesis of ligand A, metallo-building block B and Ni(ii)-α-diimine-POP. The photograph and SEM image of Ni(ii)-α-diimine-POP are inserted.

Ni(ii)-α-diimine-POP was successfully synthesized through a Sonogashira coupling reaction following the route outlined in Scheme 1. The diiodine-substituted ligand A was prepared as the bright yellow crystalline solids by double Schiff-base condensation between 4-iodo-2,6-diisopropylbenzenamine and butane-2,3-dione in good yield. The metallo-building block B was synthesized as the brick-red crystalline solids by metalation of A with DME(NiBr_2_) in moderate yield. Besides routine characterizations, the molecular structure of A was further determined by the X-ray single crystal analysis (CCDC 1906672, Fig. S1, Tables S1 and S2, ESI†). After combination of B and 1,3,5-triethynylbenzene, the Ni(ii)-α-diimine-POP was generated through Sonogashira cross-coupling reaction under solvothermal conditions (toluene, 80 °C, 72 h, 86%). After the reaction, the resulting precipitate was treated by Soxhlet extraction with CH_2_Cl_2_, methanol and acetone to remove any possible residues. After dried *in vacuo* at 110 °C for 12 h, the target POP was obtained as deep brown solids (Scheme 1, inset). The obtained POP was the irregular granular particle which was well evidenced by the scanning electron microscopy (SEM, Scheme 1, inset).

**Fig. 1:**
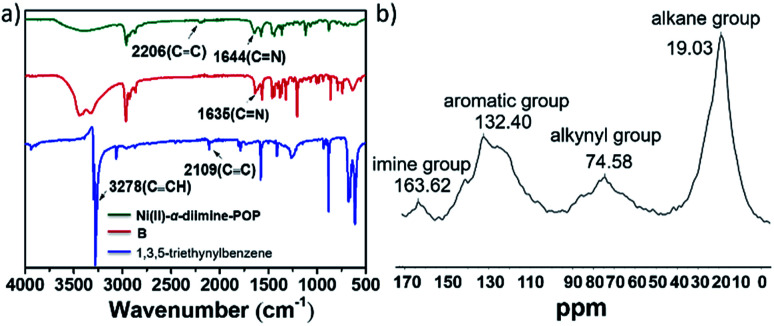
(a) IR spectra of Ni(ii)-α-diimine-POP and its precursors. (b) Solid-state ^13^C NMR of Ni(ii)-α-diimine-POP.

IR spectra (Fig. 1a) showed that the characteristic peaks at 3278 cm^−1^ attributed to the *ν*_C–H_ (–C≡C–H) in 1,3,5-triethynylbenzene basically disappeared in Ni(ii)-α-diimine-POP after coupling reaction, meanwhile the band at 2109 cm^−1^ that corresponded to the –C≡C– in 1,3,5-triethynylbenzene moved to 2206 cm^−1^, indicating that the precursors of B and 1,3,5-triethynylbenzene in POP were successfully connected to each other *via* covalent C–C bond. Furthermore, the solid-state ^13^C NMR also supported this POP formation. As indicated in Fig. 1b, the peak for the aliphatic carbon atoms of –CH_3_ and –CH(CH_3_)_2_ groups was located at 19.03 ppm.^24^ The broad signals centred at 74.58 and 132.40 ppm were respectively ascribed to the bridging –C≡C– and phenyl moieties.^25^ The signal at 163.62 ppm was associated with carbon atom in the imine group.^26^

**Fig. 2:**
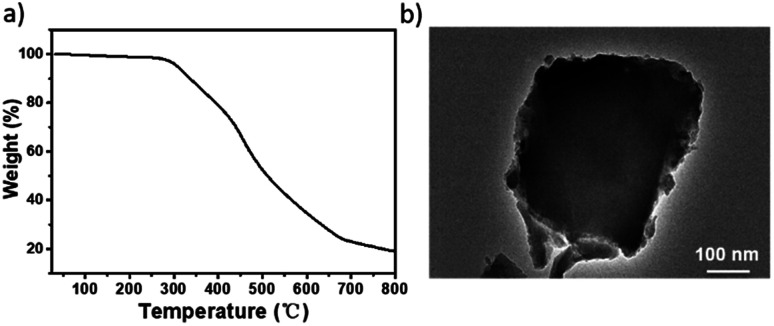
(a) TGA trace of Ni(ii)-α-diimine-POP. (b) HRTEM image of Ni(ii)-α-diimine-POP.

**Fig. 3:**
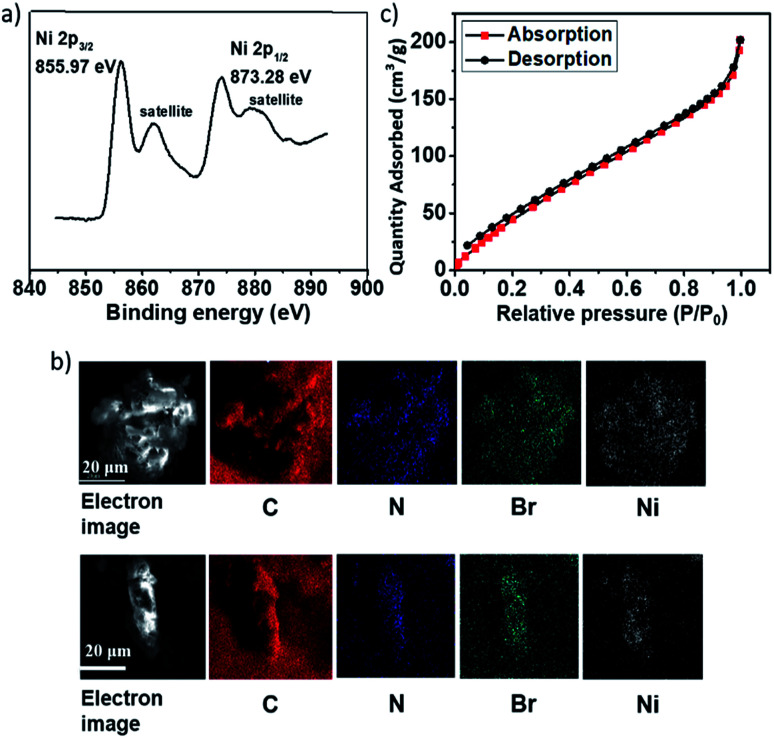
(a) XPS spectrum of Ni species in Ni(ii)-α-diimine-POP. (b) SEM-EDX spectrum of Ni(ii)-α-diimine-POP and it after five catalytic runs. (c) N_2_ sorption isotherm of Ni(ii)-α-diimine-POP.

The TGA trace indicated that the obtained Ni(ii)-α-diimine-POP remained intact till temperature over 250 °C, implying its good thermal stability (Fig. 2a). Notably, no Ni NP was generated during POP formation process, which demonstrated by the high-resolution transmission electron microscopy (HRTEM, Fig. 2b). The existed Ni species valence was demonstrated by the X-ray photoelectron spectroscopy (XPS) measurement. As shown in Fig. 3a, the observation of 2p_3/2_ and 2p_1/2_ peaks at 855.97 and 873.28 eV confirmed that the nickel species in Ni(ii)-α-diimine-POP existed as Ni(ii).^27^ The other observed doublet was attributed to intensive shake-up satellites that always occurs for Ni(ii) during acquiring XPS.^27^ On the other hand, the single N 1s peak in B (399.03 eV) and Ni(ii)-α-diimine-POP (398.96 eV) shifted to higher binding energy compared to that of A (398.88 eV), suggesting that Ni(ii) species in B and Ni(ii)-α-diimine-POP was chelated by N donors (Table 1).^28^ Inductively coupled plasma (ICP) analysis showed that the nickel content in Ni(ii)-α-diimine-POP was up to 7.6 wt% (calcd 8.3 wt%).

**Table 1:** XPS spectra of A, B, NiBr_2_, Ni(ii)-α-diimine-POP and it after five catalytic runs

Compound	N 1s_1/2_ (eV)	Ni 2p_3/2_ (eV)	Ni 2p_1/2_ (eV)
A	398.88	—	—
NiBr_2_	—	855.40	872.98
B	399.03	856.28	872.79
Ni(ii)-α-diimine-POP	398.96	855.97	873.28
Ni(ii)-α-diimine-POP after 5 runs	398.92	856.16	873.68

As mentioned above, the Ni(ii)-α-diimine-POP herein was prepared by metallo-building block *via in situ* one-pot approach, so it should feature the uniform texture. As shown in Fig. 3b, the C, N, Ni and Br species evenly distributed in the POP matrix, as evidenced by the SEM-energy dispersive X-ray (EDX) mapping.

The crystalline nature of the Ni(ii)-α-diimine-POP was characterized by PXRD measurement (Fig. S2, ESI†). A observed broad peak from 20 to 25° suggested its amorphous nature. The N_2_ sorption analysis at 77 K was used to measure its specific surface area with architectural rigidity and permanent porosity (Fig. 3c). Brunauer–Emmett–Teller (BET) analysis of Ni(ii)-α-diimine-POP indicated its surface area was up to 265.3 m^2^ g^−1^. Also, the pore size of the POP was determined based on the N_2_ sorption isotherm by employing the nonlocal density functional theory (NLDFT) method, and a series of sharp and broad peaks were observed. It was found to be mostly mesoporous having the major contribution of the pore width at *ca.* 2.7 nm (Fig. S2, ESI†).

### Catalytic properties

Next, we examined the catalytic activity of Ni(ii)-α-diimine-POP for the Suzuki–Miyaura cross coupling reactions, in which the iodobenzene and phenylboronic acid coupling was chosen as the model reaction (Fig. S3, ESI†). As shown in Table 2, the solvent screening revealed that toluene is the best one among the other solvents such as *N*,*N*-dimethylformamide (DMF), iso-propyl alcohol (IPA), dioxane, acetone, tetrahydrofuran (THF), and CH_3_CN that we tested (Table 2, entries 1 to 7). In addition, various bases, including K_3_PO_4_·3H_2_O, K_2_CO_3_, triethylamine (TEA), 1,8-diazabicyclo[5.4.0]undec-7-ene (DBU), pyridine, and piperidine, were used to performed the reaction, and we found that K_3_PO_4_·3H_2_O was better than the other inorganic and organic bases (Table 2, entries 8–13). Furthermore, when the reaction was carried out with less catalyst loading, 2 (Table 2, entry 14), 3 (Table 2, entry 15) or 4 mol% (Table 2, entry 16) instead of 5 mol%, the coupled product was obtained in significantly lower 45–89% yields. On the other hand, the reaction temperature appeared to be crucial to the catalytic efficiency. As indicated in Table 2, the catalytic activity of Ni(ii)-α-diimine-POP was significantly diminished at lower temperature (from r.t. to 80 °C, Table 2, entries 17–19). Also, shortening reaction time of 4–6 h would also lead to the significantly reduced 58–79% yields under the given reaction conditions (Table 2, entries 20–21). We did not observe any coupled product formation in the absence of the nickel source (Table 2, entry 22), further confirming the loaded Ni served as the catalytic active sites.

**Table 2:** Optimization of the model Suzuki coupling reaction between iodobenzene and phenylboronic acid^a^

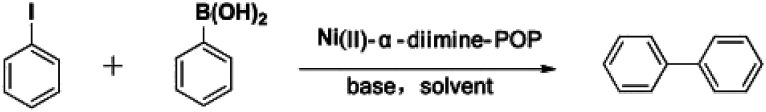						
Entry	Cat. (Ni mol% equiv.)	Base	Solvent	*T* (°C)	*T* (h)	Yield^b^ (%)
1	5	K_3_PO_4_·3H_2_O	DMF	100	8	41
2	5	K_3_PO_4_·3H_2_O	IPA	100	8	70
3	5	K_3_PO_4_·3H_2_O	Dioxane	100	8	67
4	5	K_3_PO_4_·3H_2_O	Acetone	100	8	27
**5**	**5**	**K**_**3**_ **PO**_**4**_ **·3H**_**2**_ **O**	**Toluene**	**100**	**8**	**99**
6	5	K_3_PO_4_·3H_2_O	THF	100	8	23
7	5	K_3_PO_4_·3H_2_O	CH_3_CN	100	8	8
8	5	K_3_PO_4_	Toluene	100	8	94
9	5	K_2_CO_3_	Toluene	100	8	85
10	5	TEA	Toluene	100	8	38
11	5	DBU	Toluene	100	8	12
12	5	Pyridine	Toluene	100	8	0
13	5	Piperidine	Toluene	100	8	0
14	2	K_3_PO_4_·3H_2_O	Toluene	100	8	45
15	3	K_3_PO_4_·3H_2_O	Toluene	100	8	68
16	4	K_3_PO_4_·3H_2_O	Toluene	100	8	89
17	5	K_3_PO_4_·3H_2_O	Toluene	r.t.	8	12
18	5	K_3_PO_4_·3H_2_O	Toluene	50	8	53
19	5	K_3_PO_4_·3H_2_O	Toluene	80	8	85
20	5	K_3_PO_4_·3H_2_O	Toluene	100	4	58
21	5	K_3_PO_4_·3H_2_O	Toluene	100	6	79
22	—	K_3_PO_4_·3H_2_O	Toluene	100	6	0

a Reaction conditions: iodobenzene (1.0 mmol), phenylboronic acid (1.1 mmol), base (2.0 mmol), solvent (2 mL), in N_2_.

b Yields were determined by GC analysis (Fig. S4, ESI).

Based above result, the optimized reaction conditions for the model coupling reaction were determined as: iodobenzene, 1.0 mmol; phenylboronic acid, 1.1 mmol; Ni(ii)-α-diimine-POP, 5 mol% Ni equiv.; K_3_PO_4_·3H_2_O, 2.0 mmol; temperature, 100 °C; reaction time, 8 h; toluene, 2 mL.

**Fig. 4:**
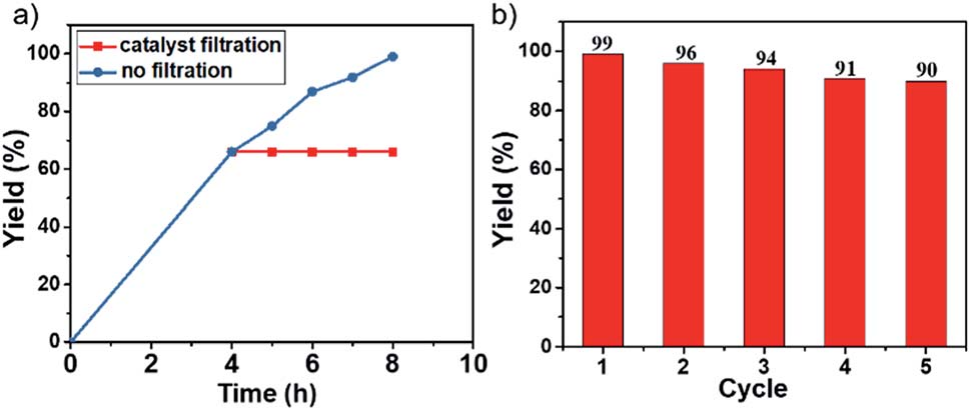
(a) Reaction time examination and leaching test for the model Suzuki cross-coupling reaction. (b) Catalytic cycle for the model Suzuki cross-coupling reaction.

To verify the heterogeneous nature of this POP-based catalyst, the hot leaching test was conducted. As shown in Fig. 4a, no further reaction occurred after ignition of the reaction at 4.0 h when it reached the yield of *ca.* 65%, at which point the heterogeneous particles were centrifuged out of the reaction mixture, and the reaction was performed for additional 4 h. GC analysis indicated that the yield of the reaction did not change during the prolonged reaction time, indicating that the Ni(ii)-α-diimine-POP exhibited a typical heterogeneous catalyst nature herein.

Once we confirmed the heterogeneous nature of the catalyst, we also tested its recyclability. After each catalytic run, the solid catalyst was separated by centrifugation, washed with toluene (3 × 2 mL), CH_3_CN (3 × 2 mL), and dried at 110 °C for 12 h in vacuum. The recycling catalytic runs were conducted by combining the recovered catalyst with inorganic base, iodobenzene, and phenylboronic acid in toluene. As shown in Fig. 4b, the solid catalyst of Ni(ii)-α-diimine-POP still showed excellent activity and the cross-coupling yield was even up to 90% after five catalytic cycles (Fig. S5, ESI†). After multiple catalytic cycles, ICP measurement demonstrated that the Ni content in Ni(ii)-α-diimine-POP was 6.3 wt%, suggesting a 17% Ni leaching occurred after five catalytic runs. This catalyst loss might be responsible for this slight yield decrease. On the other hand, no valence change for Ni species was observed (Table 1), implying that the Ni species in POP was stable during the reusable processes under the given conditions. In addition, the POP morphology and elemental distribution were well maintained after the recycle (Fig. 3b and S6†).

To test the available scope of this catalyst, we performed coupling reactions of phenylboronic acid with a series of substituted aryl halides under optimized conditions (Table 3). It is noteworthy that the catalytic system was tolerant to a wide range of functional groups such as –CF_3_, –NO_2_, –COCH_3_, –CH_3_, –OCH_3_, –CO_2_Me, and –CN at different substituted positions. Aryl bromides with both electron-donating and electron-withdrawing groups at *para*-, *meta*- or *ortho*-substituted position afforded cross-coupling products of i–viii (Table 3, entries 1–9) with good-to-excellent yields (90–98%). Compared to aryl bromides, the aryl iodides for the cross-coupling reaction were more active. As shown in Table 3 (entries 10–15), the yields for the target coupled products of i, iv, v and viii–x based on iodobenzene and *para*- or *meta*-substituted aryl iodides were more than 99%. Meanwhile, the *ortho*-substituted iodobenzene provided the products of xi and xii in slightly lower 90–92% yields (Table 3, entries 16 and 17). On the other hand, the crossing-coupling reactions between aryl iodide and phenylboronic acids with both electron- donating and electron-withdrawing groups at *para*-, *meta*- or *ortho*-substituted position still afforded excellent 98 to >99% yields (Table 3, entries 18–22, compounds iii, v, vii, xiii and xiv). The slightly lower 90% yield for iv from *para*-methoxyphenylboronic acid with aryl iodide was also observed (Table 3, entry 23). However, the chlorobenzene and phenylboronic acid coupling gave a low 26% yield of i in 12 h (Table 3, entry 24), indicating that the Ni(ii)-α-diimine-POP cannot significantly activate the ArCl-based Suzuki–Miyaura cross-coupling reactions.

**Table 3:** Cross-coupling reactions of various aryl halides and aryl boric acids catalysed by Ni(ii)-α-diimine-POP^a^

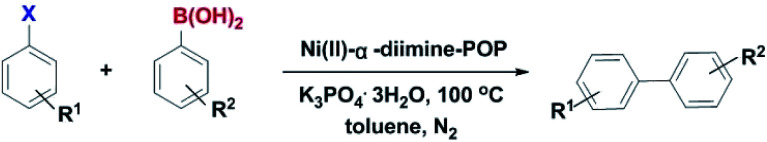						
Entry	R^1^	X	R^2^	*t* (h)	Product	Yield^b^ (%)
1	H	Br	H	12	i	94
2	4-NO_2_	Br	H	12	ii	97
3	4-COCH_3_	Br	H	12	iii	98
4	4-OCH_3_	Br	H	12	iv	92
5	4-CO_2_CH_3_	Br	H	12	v	90
6	2-CN	Br	H	12	vi	90
7	2-CH_3_	Br	H	12	vii	93
8	3-NO_2_	Br	H	12	viii	92
9	H	Br	4-OCH_3_	12	iv	90
10	H	I	H	8	i	99
11	3-CH_3_	I	H	8	ix	>99
12	3-NO_2_	I	H	8	viii	>99
13	4-OCH_3_	I	H	8	iv	>99
14	4-CN	I	H	8	x	>99
15	4-CO_2_CH_3_	I	H	8	v	>99
16	2-OCH_3_	I	H	8	xi	92
17	2-CF_3_	I	H	8	xii	90
18	3-NO_2_	I	4-F	8	xiii	99
19	4-CN	I	3-OCH_3_	8	xiv	>99
20	H	I	2-CH_3_	8	vii	>99
21	H	I	4-COCH_3_	8	iii	98
22	H	I	4-CO_2_CH_3_	8	v	98
23	H	I	4-OCH_3_	8	iv	90
24	H	Cl	H	12	i	26

a Reaction conditions: aryl halide (1.0 mmol), arylboronic acid (1.1 mmol), K_3_PO_4_·3H_2_O (2 mmol, 0.533 g), Ni(ii)-α-diimine-POP (5 mol% Ni equiv.), toluene (2 mL), 100 °C, N_2_.

b Yields were determined by GC analysis (Fig. S7, ESI).

Compared to the reported Ni-loaded solid catalytic systems (Table 4), which usually contained coexistent such as PPh_3_ or other metal oxides, for the Suzuki–Miyaura coupling reaction, it exhibited an impressive catalytic performance. On the other hand, we believe the Ni-catalysed Suzuki–Miyaura cross-coupling reaction herein went through the same mechanism as the reported one,^33–35^ which should involve the initial oxidative addition of the aryl halide to a Ni(0) species, followed by trans-metalation and subsequent reductive elimination step to afford the expected coupling product, meanwhile the catalytically active nickel(0) species was regenerated (Fig. S8, ESI†).

**Table 4:** Comparison of Ni(ii)-α-diimine-POP with the reported Ni-loaded solid catalysts for the Suzuki cross-coupling reaction between iodobenzene/bromotoluene and arylboronic acid

Cat. (mol%)	Conditions	Substrate/yield (%)	Run	Ref.
Ni/C_g_ (8)	PPh_3_/dioxane/THF, *n*-BuLi/KF/LiOH, reflux, 9 h	4-BrPhMe/PhB(OH)_2_/87	2	33
Ni-PVP/TiO_2_–ZrO_2_ (20)	MeOH/H_2_O, K_2_CO_3_, 60 °C, 8 h	PhI/PhB(OH)_2_/97	6	34
UiO-66(L1)/NiCl_2_/PPh_3_ (3)	CH_3_CN, K_2_CO_3_, 65 °C, 12 h	PhBr/PhB(OH)_2_/71	—	35
UiO-66(L2)/NiCl_2_/PPh_3_ (3)	CH_3_CN, K_2_CO_3_, 65 °C, 12 h	PhBr/PhB(OH)_2_/84	—	35
UiO-66(L3)/NiCl_2_/PPh_3_ (3)	CH_3_CN, K_2_CO_3_, 65 °C, 12 h	PhBr/PhB(OH)_2_/90	—	35
UiO-66(L3)/Ni(COD)_2_/PPh_3_ (3)	CH_3_CN, K_2_CO_3_, 65 °C, 12 h	PhBr/PhB(OH)_2_/96	7	35
Ni(ii)-α-diimine-POP (5%)	Toluene, K_3_PO_4_·3H_2_O, 100 °C, 8 h	PhI/PhB(OH)_2_/99	5	This work
Ni(ii)-α-diimine-POP (5%)	Toluene, K_3_PO_4_·3H_2_O, 100 °C, 12 h	PhBr/PhB(OH)_2_/94	5	This work

## Experimental

### Materials and measurements

All chemicals and solvents were at least of analytic grade and employed as received without further purification. The elemental analysis was conducted on a PerkinElmer Model 2400 analyzer. MS spectra were obtained by Bruker maxis ultra-high resolution-TOF MS system. NMR data were collected using an AM-400 spectrometer. ^13^C CP/MAS NMR experiments were performed on Agilent 600 DD2 spectrometer at a resonance frequency of 150.15 MHz. ^13^C NMR spectra were recorded on a spinning rate of 15 kHz with a 4 mm probe at room temperature. ^13^C CP/MAS experiments were performed with a delay time of 5 s and a contact time of 1 ms. Infrared spectra were obtained in the 400–4000 cm^−1^ range using a Bruker ALPHA FT-IR spectrometer. Powder X-ray diffraction (PXRD) measurements were performed at 293 K on a D8 ADVANCE diffractometer (Cu Kα, *λ* = 1.5406 Å). ICP analysis was performed on an IRIS InterpidII XSP and NU AttoM. XPS spectra were obtained from PHI Versaprobe II. Thermogravimetric analyses were carried out on a TA Instrument Q5 simultaneous TGA under flowing nitrogen at a heating rate of 10°C min^−1^. HRTEM (high resolution transmission electron microscopy) analysis was performed on a JEOL 2100 Electron Microscope at an operating voltage of 200 kV. The scanning electron microscopy (SEM) micrographs were recorded on a Gemini Zeiss Supra TM scanning electron microscope equipped with energy-dispersive X-ray detector (EDX). The elemental analysis was conducted on a PerkinElmer Model 2400 analyzer. The crystal data were obtained by Agilent SuperNova X-ray single crystal diffractometer.

### Synthesis of A^29^

A mixture of 2,6-diisopropylbenzenamine (2.54 g, 14.3 mmol) and I_2_ (4 g, 15.7 mmol) was charged in 50 mL round-bottom flask. Then, 10 mL cyclohexane and 4 mL saturated Na_2_CO_3_ solution added in succession. After stirred at room temperature for 12 h, the mixture was diluted with EtOAc (20 mL) and washed with saturated Na_2_S_2_O_3_ (3 × 40 mL). The combined organic layer was dried with anhydrous MgSO_4_. The crude product was purified by column chromatography (petroleum ether/EtOAc = 10/1) to give 4-iodo-2,6-diisopropylbenzenamine as a black liquid (3.90 g, 96%). ^1^H NMR (400 MHZ, CDCl_3_) *δ*: 7.37 (s, 2H), 3.82 (s, 2H), 2.94 (2m, 2H), 1.37 (m, *J* = 8 Hz, 12 H); ^13^C NMR (400 MHz, CDCl_3_), *δ*: 22.4 (4c), 28.0 (2c), 81.2, 131.8 (2c), 135.1 (2c), 140.2.; IR (KBr): 3486 (w), 3404 (w), 2961 (vs), 2870 (m), 1618 (s), 1570 (w), 1460 (s), 1438 (vs), 1384 (m), 1364 (m), 1350 (m), 1299 (w), 1250 (m), 1208 (m), 1125 (w), 1061 (w), 924 (w), 865 (m), 832 (w), 765 (w), 746 (w), 716 (w), 555 (w). HRMS (ESI-TOF) calcd for C_12_H_19_IN ([M + H]^+^), *m*/*z* 304.0517; found, *m*/*z* 304.0592.

A mixture of 4-iodo-2,6-diisopropylbenzenamine (3.7 g, 14 mmol), butane-2,3-dione (0.603 mL, 7 mmol) and 0.5 mL formic acid in 15 mL methanol was charged in 50 mL Schlenk flask. After stirred at room temperature for 6 h, the precipitate was filtered and recrystallized from ethanol to afford A as a bright yellow solid (4.4 g, 81%). ^1^H NMR (400 MHz, CDCl_3_) *δ* 7.46 (s, 2H), 2.63 (m, 2H), 2.07 (s, 3H), 1.18 (d, 12H); ^13^C NMR (400 MHz, CDCl_3_) *δ* 168.5 (2c), 145.8 (2c), 137.8 (2c), 137.8 (2c), 132.3 (4c), 88.3 (2c), 28.5 (2c), 22.8 (8c), 22.5 (4c), 16.7 (2c). IR(KBr): 3486(w), 3403(w), 2961(vs), 2870(m), 1648(s), 1570(w), 1460(s), 1438(s),1384(w), 1364(s), 1324(m), 1242(s), 1186(s), 1126(s), 1072(w), 937(m), 883(w), 864(w), 819(w), 792(m), 711(m), 638(w), 559(w), 539(w), 467(w). HRMS (ESI-TOF) calcd for C_28_H_39_I_2_N_2_ ([M + H]^+^), *m*/*z* 658.1191; found, *m*/*z* 658.1206.

### Synthesis of B^30,31^

A mixture of A (1.1 mmol, 0.722 g) and DME(NiBr_2_)^32^ (1 mmol, 0.308 g) in 50 mL dry dichloromethane was charged in a 100 mL Schlenk flask. After stirred at room temperature for 2 day in N_2_, the reaction system was filtered though a pad of Celite. The resulting solids were further washed with anhydrous diethyl ether, and then dried *in vacuo* to afford complex B as brick-red solid (0.59 g, 68.0%). IR(KBr): 3439(s), 3326(s), 2965(s), 2929(m), 2868(w), 1636(w), 1567(m), 1462(m), 1441(m), 1408(w), 1382(m), 1364(w), 1346(w), 1323(m), 1306(w), 1256(w), 1240(w), 1207(s), 1168(w), 1153(w), 1069(w), 1006(w), 985(w), 940(w), 887(w), 863(m), 791(m), 742(w), 636(w), 554(w), 480(w). Anal. calcd: for C_28_H_38_Br_2_I_2_NiN_2_, C 37.92, H 4.61, N 3.17, Br 17.31. I 30.4%, Ni 6.6% (I and Ni wt% were determined by ICP measurement).

### Synthesis of Ni(ii)-α-diimine-POP

A mixture of the compound B (0.5 mmol, 0.488 g), 1,3,5-triethynylbenzene (0.5 mmol, 75 mg), Pd(PPh_3_)_4_ (0.25 mmol, 30 mg), CuI (0.1 mmol, 20 mg) and triethylamine (25 mL) in 50 mL toluene was heated at 80 °C for 72 h in N_2_. After cooled to room temperature, the obtained crude product was completely washed with water (30 mL), ethanol (30 mL) and dichloromethane (30 mL) respectively. The resulted solids were further Soxhlet extracted with mixed solution of dichloromethane, methanol and acetone (50 mL: 50 mL: 50 mL) and then dried at 110 °C *in vacuo* to afford Ni(ii)-α-diimine-POP as dark gray solids (0.31 g, 86.4%). IR(KBr): 3390(s), 3030(w), 2924(vs), 2852(s), 2041(w), 1705(s), 1595(m), 1518(w), 1492(m), 1463(s), 1377(m), 1225(m), 1085(m), 1015(m), 830(m), 749(w), 616(w). Anal. calcd: C 58.2, H 4.93, N 3.11, Br 19.62. I 5.67%, Ni 7.6% (I and Ni wt% were determined by ICP measurement).

### General procedure for the Suzuki–Miyaura cross-coupling reaction between iodobenzene and arylboronic acid

A mixture of iodobenzene (1.0 mmol, 116 μL), phenylboronic acid (1.1 mmol, 0.134 g), K_3_PO_4_·3H_2_O (2 mmol, 0.533 g) and Ni(ii)-α-diimine-POP (52 mg) in 2 mL toluene was stirred at 100 °C for 8 or 12 h in N_2_ to afford the corresponding product. Yield was determined by the GC analysis.

### General procedure for the Suzuki–Miyaura cross-coupling reaction between bromobenzene and arylboronic acid

A mixture of bromobenzene (1.0 mmol, 104 μL), phenylboronic acid (1.1 mmol, 0.134 g), K_3_PO_4_·3H_2_O (2 mmol, 0.533 g) and Ni(ii)-α-diimine-POP (52 mg) in 2 mL toluene was stirred at 100 °C for 8 h or 12 h in N_2_ to afford the corresponding product. Yield was determined by the GC analysis.

### General procedures for the recycle of Ni(ii)-α-diimine-POP

After each catalytic run, the solid catalyst was recovered by centrifugation, washed with toluene (3 × 2 mL), acetonitrile (3 × 2 mL) and dried at 110 °C for 12 h in vacuum and then was reused for the next catalytic run under the same reaction conditions.

## Conclusions

In summary, we report herein a new Ni(ii)-α-diimine decorated porous organic polymer Ni(ii)-α-diimine-POP by assembly of metallo-building block and its polymerized partner *via in situ* one-pot approach. The resulting polymer is porous, solvent- and thermal-stable. More importantly, the obtained Ni(ii)-α-diimine-POP can highly promote the Suzuki–Miyaura cross-coupling reaction in a heterogeneous way with excellent yields and a reasonable scope. The catalyst could be reused at least 5 times without significant loss of the catalytic activity (>90% yield). We expect the presented approach to be viable for the construction of many more new metalated POP-based heterogeneous catalytic materials for various organic transformations.

## Conflicts of interest

There are no conflicts to declare.

## Supplementary Material

RA-009-C9RA03679B-s001

RA-009-C9RA03679B-s002
