# Supplementary material for: Nickel-metalated porous organic polymer for Suzuki–Miyaura cross-coupling reaction
Source: RSC Adv. 2019 Jun 28;9(35):20266–72. doi: 10.1039/c9ra03679b (PMC9065688; doi:10.1039/c9ra03679b)
Supplement: RA-009-C9RA03679B-s001 [file RA-009-C9RA03679B-s001.pdf]

Electronic Supplementary Material (ESI) for RSC Advances.
This journal is © The Royal Society of Chemistry 2019

Supporting information for

Nickel-metalated porous organic polymer for Suzuki-Miyaura cross-coupling reaction

Ying Dong, Jing-Jing Jv, Yue Li, Wen-Han Li, Yun-Qi Chen, Qian Sun, Jian-Ping Ma, Yu-Bin Dong*

College of Chemistry, Chemical Engineering and Materials Science, Collaborative Innovation Center of Functionalized Probes for Chemical Imaging in Universities of Shandong, Key Laboratory of Molecular and Nano Probes, Ministry of Education, Shandong Normal University, Jinan 250014, P. R. China. E-mail: yubindong@sdnu.edu.cn

Single-crystal data of A.

X-ray single-crystal analysis of **A** were measured at room temperature (298 K) on a Bruker SMART APEX CCD-based diffractometer (Cu Kα radiation, λ = 1.52 A).[1,2] After determination of crystal quality and initial triclinic unit cell parameters, a hemi sphere of frame data was collected. The raw data frames were integrated with SAINT+,[3,4] which also applied corrections for Lorentz and polarization effects. The final unit cell parameters are based on the least-squares refinement of 4862 reflections from the data set with I > 5σ (I). Analysis of the data showed negligible crystal decay during data collection. The structure was solved by a combination of direct methods and difference Fourier syntheses, and refined by full-matrix least-squares against F^2, using the SHELXTL software package.[3,4] All non-hydrogen atoms of the framework were refined with anisotropic displacement parameters. The details of crystallographic data and structure refinement parameters are summarized in Table S1. The selected bonds lengths and angles for **A** were shown in Table S2. Crystallographic data (excluding structure factors) for the structure reported in this paper have been deposited with the Cambridge Crystallographic Data Center as supplementary publication no CCDC 1906672. Copies of the data can be obtained free of charge on application to CCDC, 12 Union Road, Cambridge CB2 1EZ, UK (fax: (+44)1223-336-033; e-mail: deposit@ccdc.cam.ac.uk).

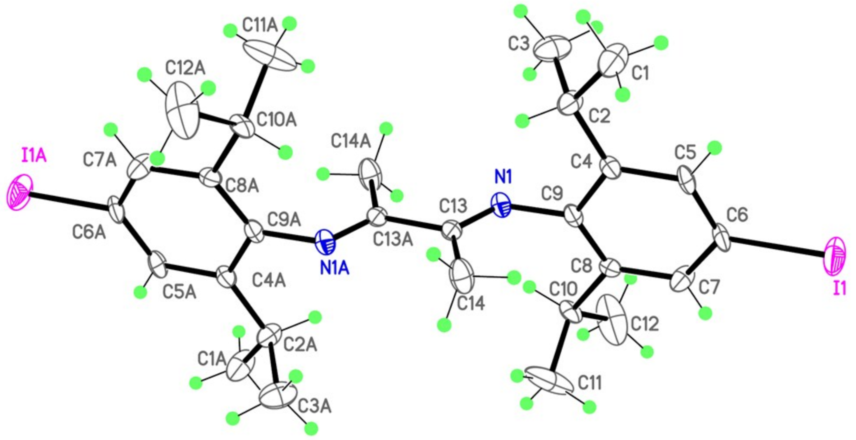

Fig. S1 The ORTEP figure of **A**.

Table S1. Crystal data and structure refinement for A

Complexes	A
Empirical formula	$C_{28}H_{38}N_2I_2$
Formula weight	656.4
Temperature	200.00(10) K
Crystal system	monoclinic
Space group	$P2_1$/c
Unit cell dimensions	a=9.6628(3) Å
	b=17.8319(5) Å beta=105.541(3) deg
	c=8.5916(3) Å
Volume	1426.26(8) Å^3
Z,	2
Calculate density	1.528 Mg/m^3
Absorption coefficent	17.429 mm^{-1}
F(000)	652
Crystal size	0.29 × 0.13 × 0.02 mm^3
2Θ range for data collection	9.5 to 134.04 deg
Index ranges	-11 ≤ h ≤ 10, -21 ≤ k ≤ 21, -7 ≤ l ≤ 10
Reflections collected	5003
Independent reflections	2547[R(int) = 0.0861]
Data/restraints/parameters	2547/0/150
Goodness-of-fit on F^2	1.098
Final R indexes [I>=2σ (I)]	R_1= 0.0902, wR_2= 0.2424
Final R indexes [all data]	R_1= 0.0926, wR_2= 0.2483
Largest diff. peak and hole	2.32/-2.20 e Å^{-3}

Table S2. Selected bond lengths and angles for A

Bonds lengths	C(1)-C(2) 1.528(8)	C(10)-C(12) 1.540(10)
	C(2)-C(3) 1.545(11)	C(13)-C(13) 1.505(12)
	C(2)-C(4) 1.507(9)	C(13)-C(14) 1.503(9)
	C(4)-C(5) 1.405(9)	C(13)-N(1) 1.283(8)
	C(4)-C(9) 1.406(9)	
	C(5)-C(6) 1.394(10)	
	C(6)-C(7) 1.380(9)	
	C(6)-I(1) 2.078(7)	
	C(7)-C(8) 1.391(10)	
	C(8)-C(9) 1.408(9)	
	C(8)-C(10) 1.526(9)	
	C(9)-N(1) 1.404(8)	
	C(10)-C(11) 1.483(11)	

bond angles	C(1)-C(2)-C(3)	108.7(6)	C(7)-C(8)-C(10)	121.0(6)
	C(4)-C(2)-C(11)	14.2(5)	C(9)-C(8)-C(10)	119.6(6)
	C(4)-C(2)-C(3)	110.2(6)	C(4)-C(9)-C(8)	120.7(6)
	C(5)-C(4)-C(2)	122.3(6)	N(1)-C(9)-C(4)	119.7(6)
	C(5)-C(4)-C(9)	117.7(6)	N(1)-C(9)-C(8)	119.2(6)
	C(9)-C(4)-C(2)	120.0(6)	C(8)-C(10)-C(12)	111.2(5)
	C(6)-C(5)-C(4)	121.5(5)	C(11)-C(10)-C(8)	114.1(6)
	C(5)-C(6)-I(1)	118.9(4)	C(11)-C(10)-C(12)	109.9(8)
	C(7)-C(6)-C(5)	119.5(6)	C(14)-C(13)-C(13)	1118.3(7)
	C(7)-C(6)-I(1)	121.6(5)	N(1)-C(13)-C(13)	1115.4(7)
	C(6)-C(7)-C(8)	120.9(6)	N(1)-C(13)-C(14)	126.3(6)
	C(7)-C(8)-C(9)	119.3(6)	C(13)-N(1)-C(9)	120.0(6)

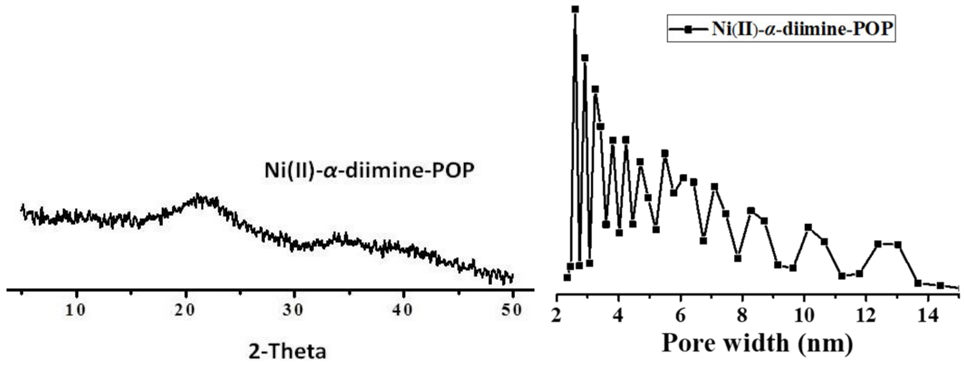

Fig. S2 Left: PXRD pattern of **Ni(II)-α-diimine-POP**, suggesting its amorphous nature. Right: Pore size distribution calculated by NL-DFT.

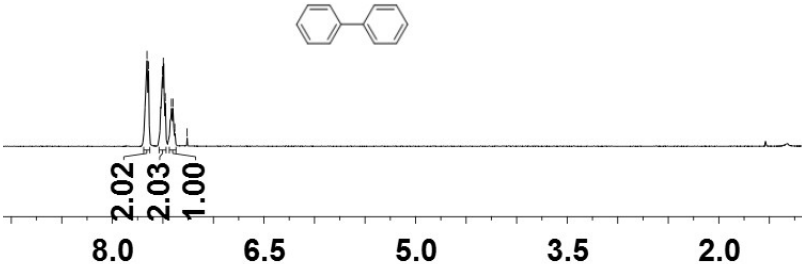

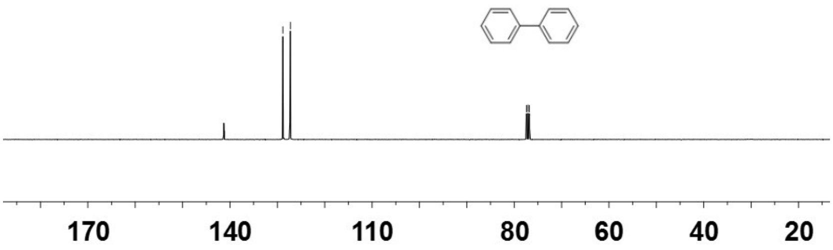

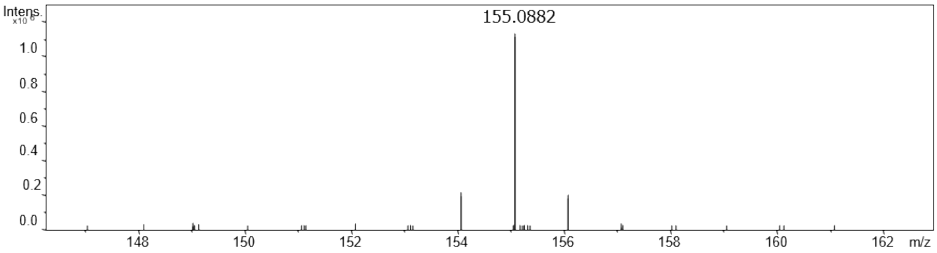

Fig. S3 ^{1}H NMR (400 MHz, $CDCl_3$) δ = 7.64-7.66 (d, *J* = 8 Hz, 4H), 7.48-7.50 (m, 4H), 7.38-7.42 (m, 2H). ^{13}C NMR (400 MHz, $CDCl_3$) δ 141.1 (2c), 128.8 (4c), 127.3 (2c), 127.2 (4c). ESI-MS (ESI-MS: calcd for $C_{12}H_{10}$, 155.0855 ([M+H$^+$]); found: *m/z* 155.0882) spectrum for the product generated from the model Suzuki-Miyaura cross-coupling reaction.

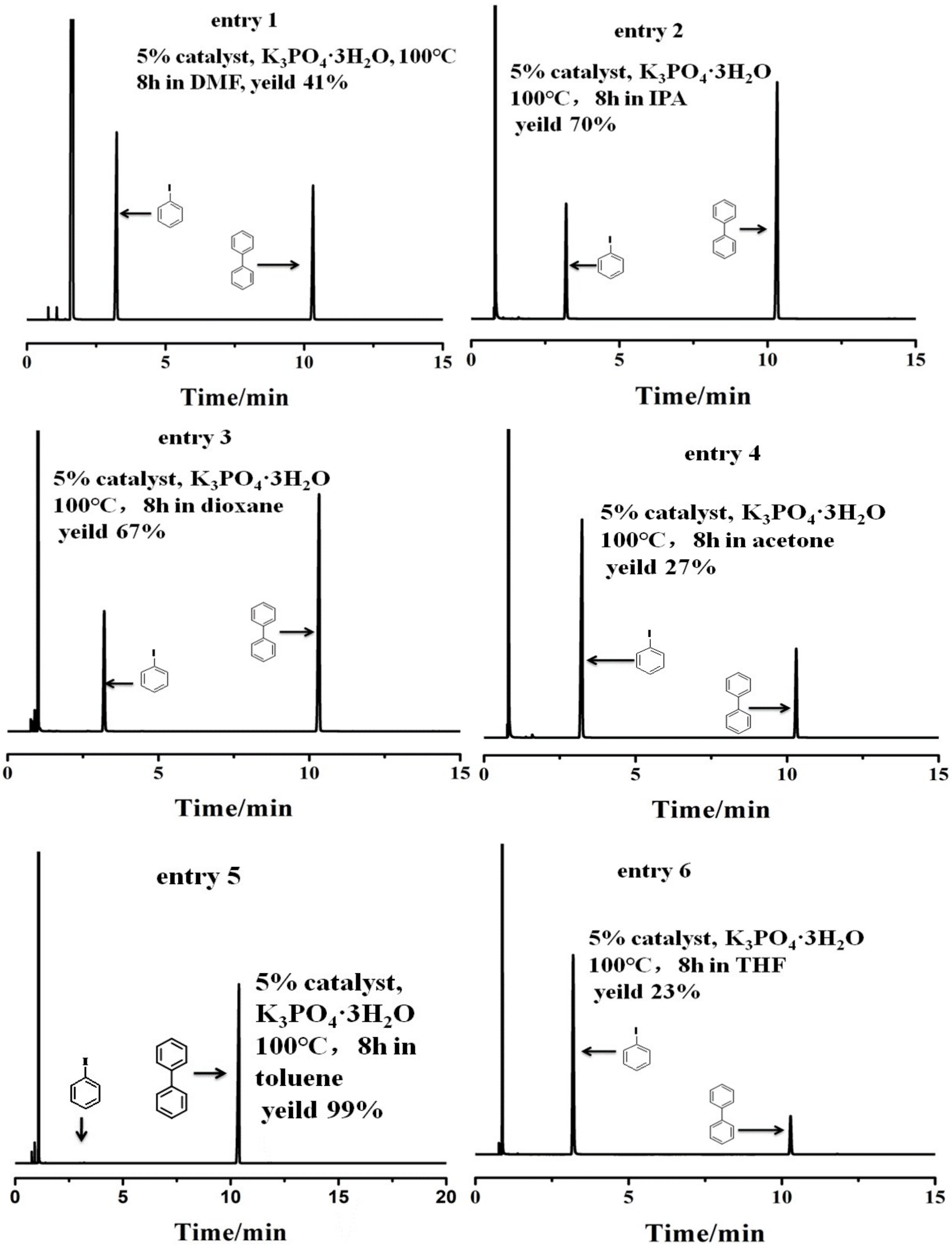

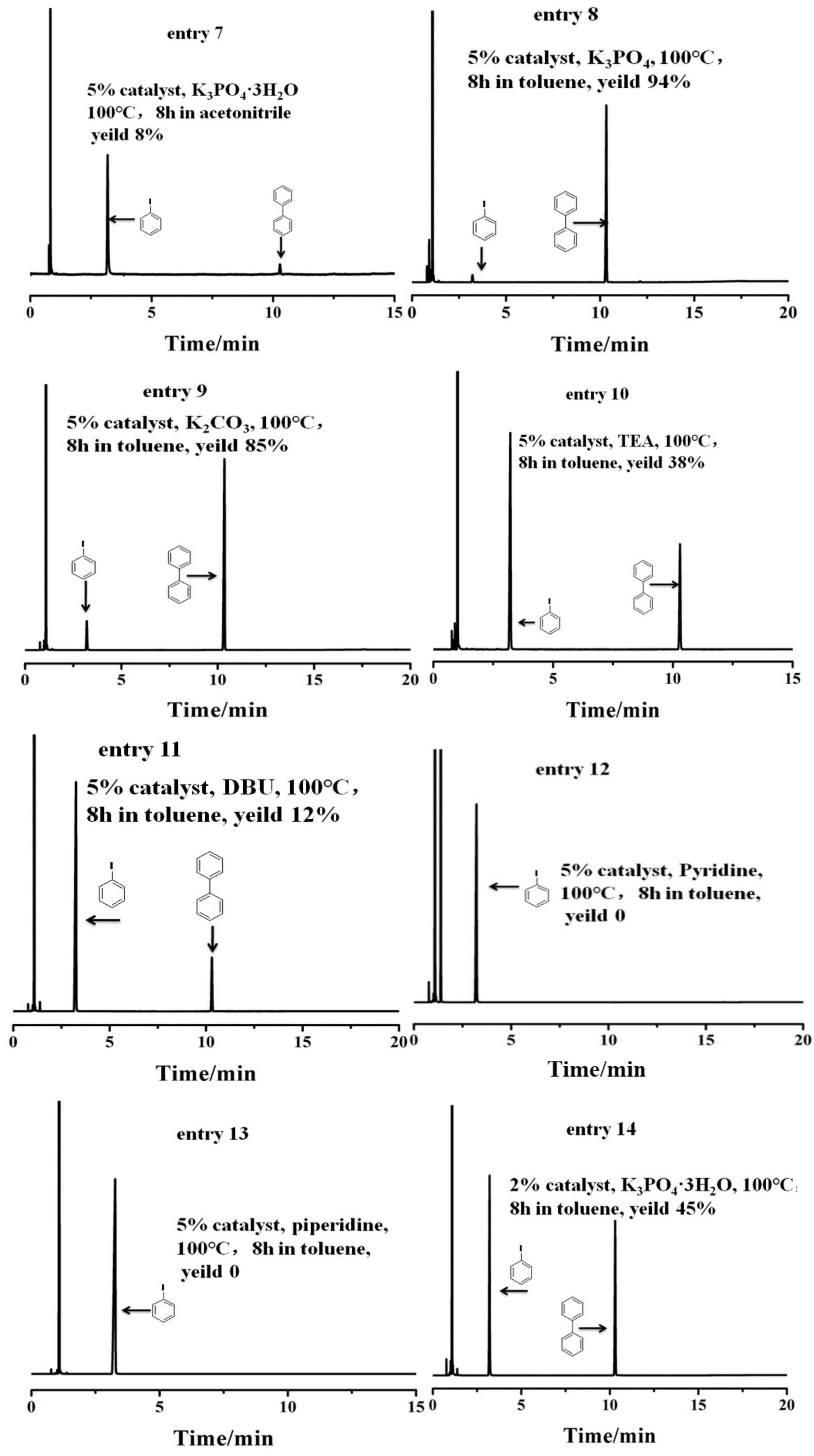
entry 7
5% catalyst, K3PO4·3H2O
100°C， 8h in acetonitrile
yeild 8%
Time/min
entry 8
5% catalyst, K3PO4, 100°C，
8h in toluene, yeild 94%
Time/min
entry 9
5% catalyst, K2CO3, 100°C，
8h in toluene, yeild 85%
Time/min
entry 10
5% catalyst, TEA, 100°C，
8h in toluene, yeild 38%
Time/min
entry 11
5% catalyst, DBU, 100°C，
8h in toluene, yeild 12%
Time/min
entry 12
5% catalyst, Pyridine,
100°C， 8h in toluene,
yeild 0
Time/min
entry 13
5% catalyst, piperidine,
100°C， 8h in toluene,
yeild 0
Time/min
entry 14
2% catalyst, K3PO4·3H2O, 100°C
8h in toluene, yeild 45%
Time/min

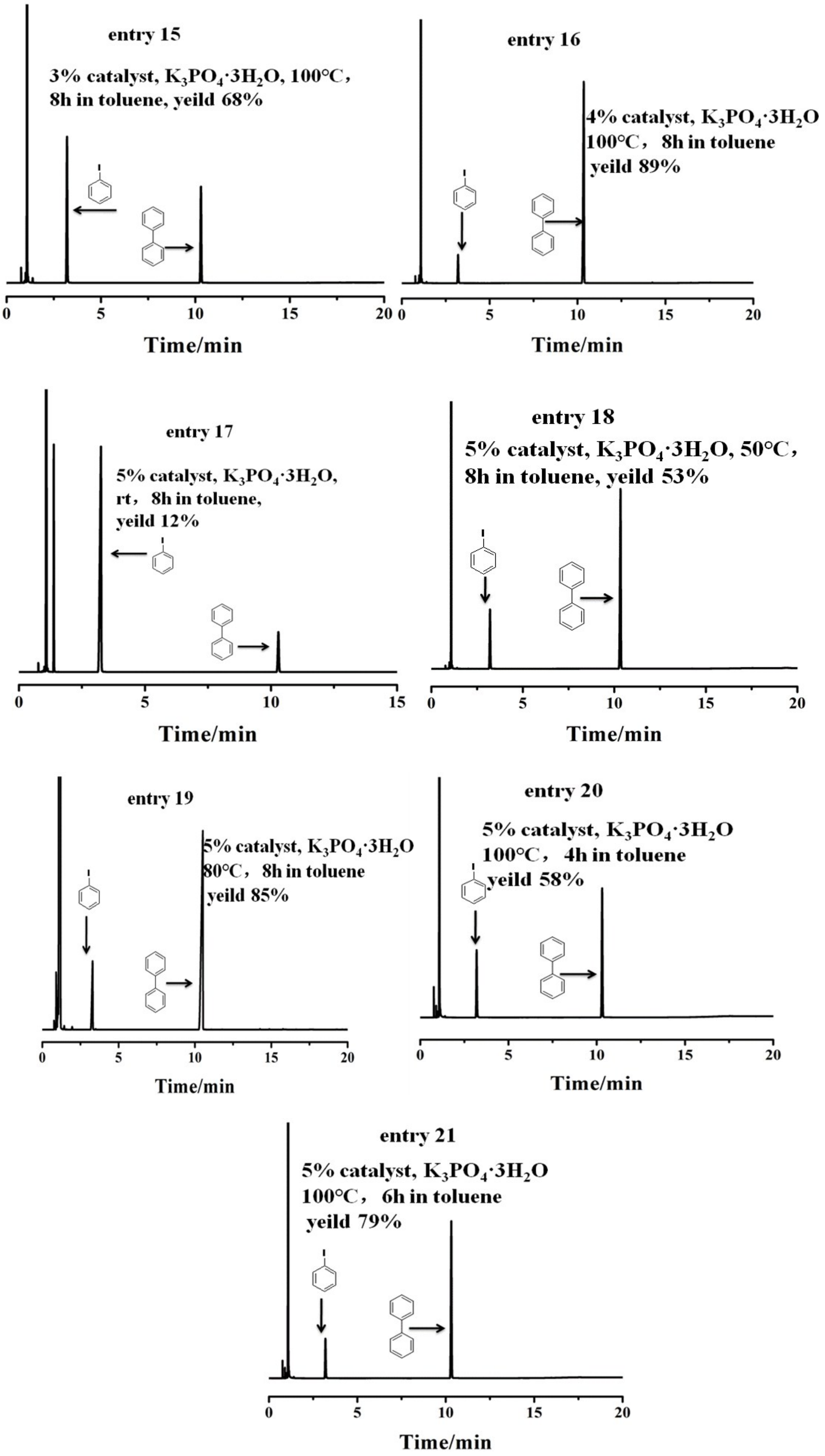

Fig. S4 GC analysis of the model Suzuki-Miyaura cross-coupling reaction catalyzed by **Ni(II)-*α*-diimine-POP** under different reaction conditions (for Table 1).

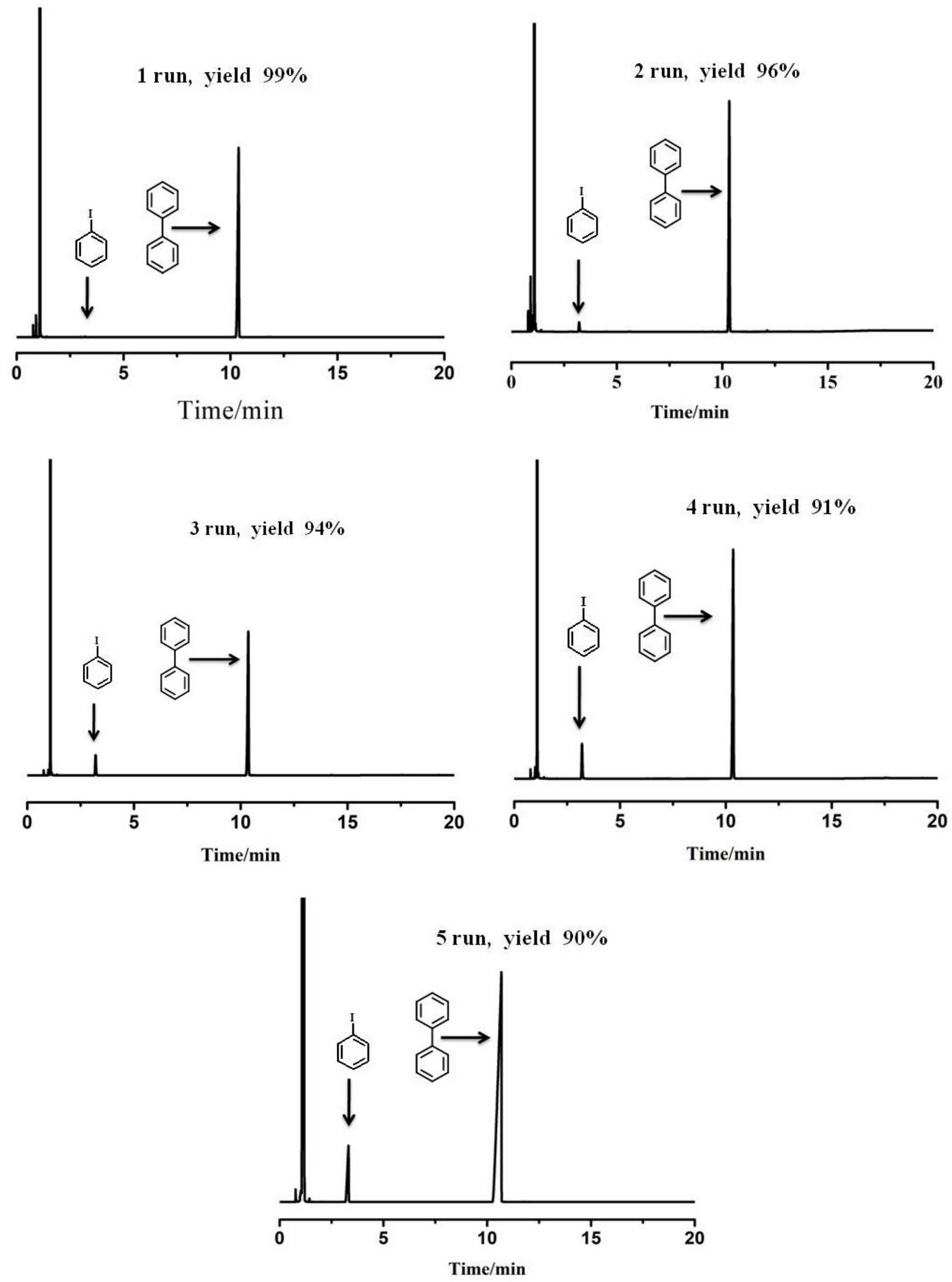

Fig. S5 GC analysis for the model Suzuki cross-coupling reaction catalysed by **Ni(II)-α-diimine-POP** for 5 catalytic runs.

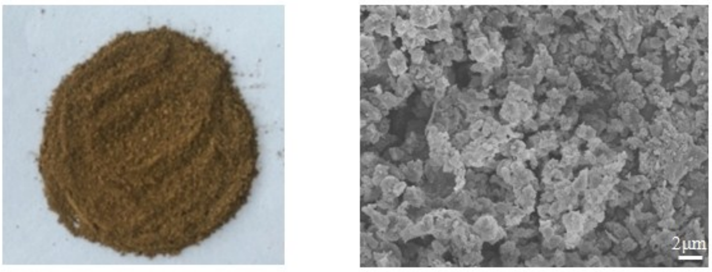

Fig. S6 Ni(II)-α-diimine-POP sample (left), SEM (right) after five catalytic runs.

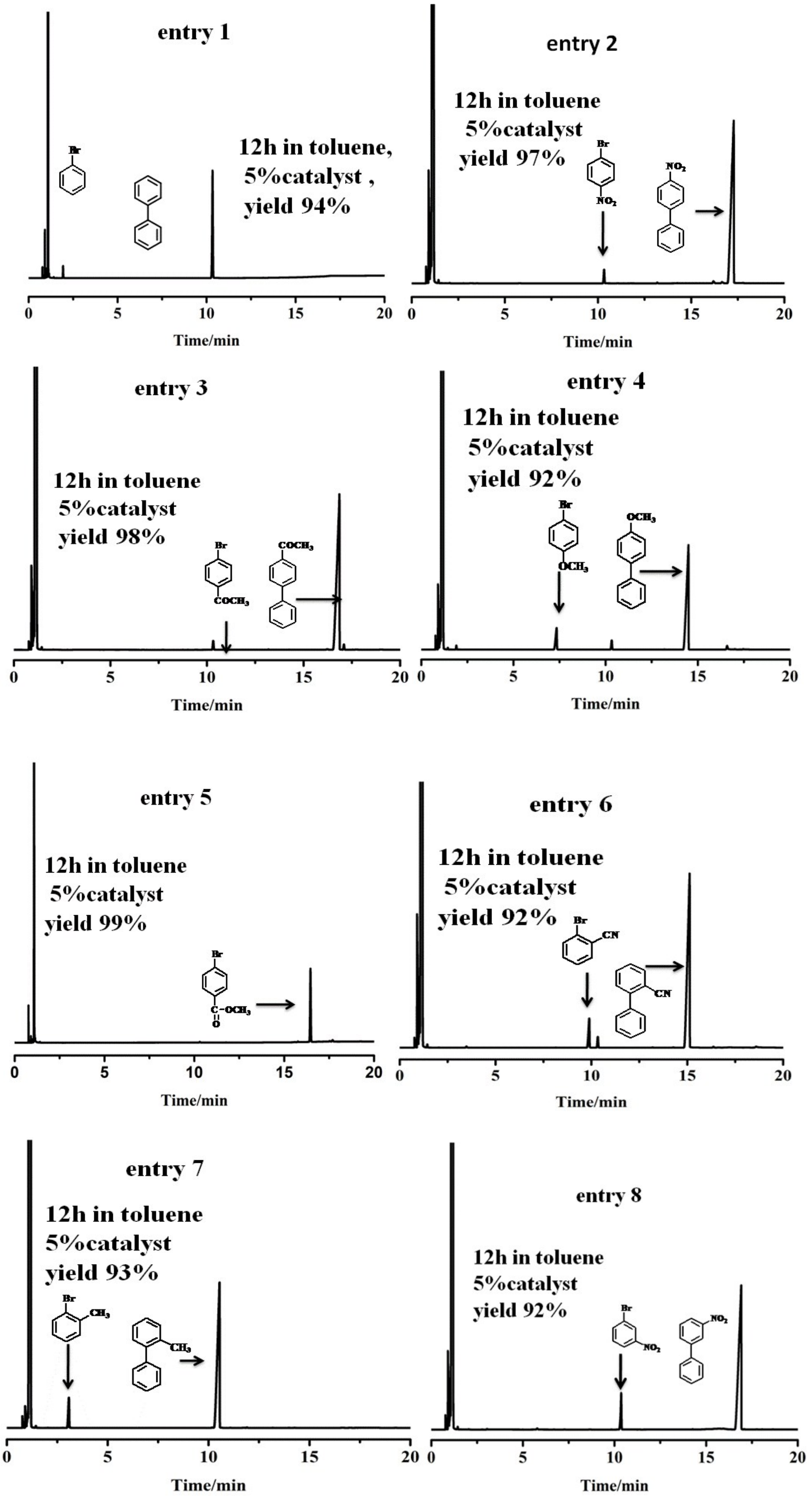
entry 1
12h in toluene,
5%catalyst ,
yield 94%
Br
0 5 10 15 20
Time/min
entry 2
12h in toluene
5%catalyst
yield 97%
Br
NO₂
NO₂
0 5 10 15 20
Time/min
entry 3
12h in toluene
5%catalyst
yield 98%
Br
COCH₃
COCH₃
0 5 10 15 20
Time/min
entry 4
12h in toluene
5%catalyst
yield 92%
Br
OCH₃
OCH₃
0 5 10 15 20
Time/min
entry 5
12h in toluene
5%catalyst
yield 99%
Br
C-OCH₃
O
0 5 10 15 20
Time/min
entry 6
12h in toluene
5%catalyst
yield 92%
Br
CN
CN
0 5 10 15 20
Time/min
entry 7
12h in toluene
5%catalyst
yield 93%
Br
CH₃
CH₃
0 5 10 15 20
Time/min
entry 8
12h in toluene
5%catalyst
yield 92%
Br
NO₂
NO₂
0 5 10 15 20
Time/min

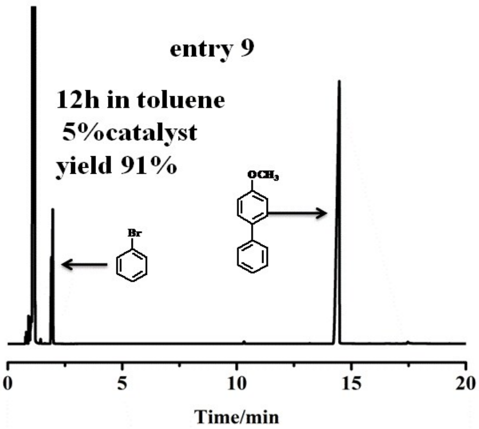
entry 9
12h in toluene
5%catalyst
yield 91%
0 5 10 15 20
Time/min

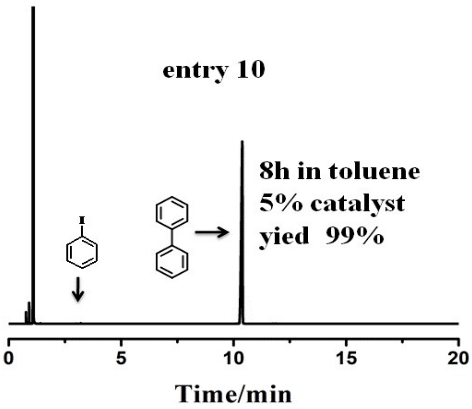
entry 10
8h in toluene
5% catalyst
yied 99%
0 5 10 15 20
Time/min

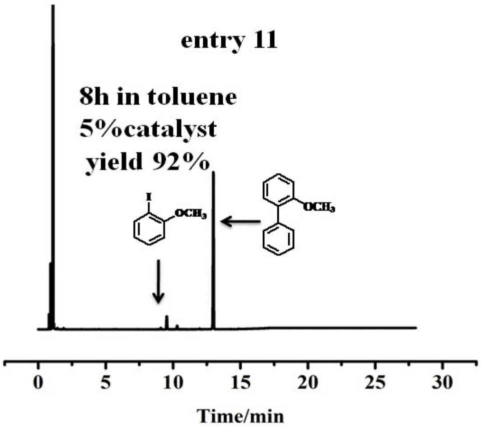
entry 11
8h in toluene
5%catalyst
yield 92%
0 5 10 15 20 25 30
Time/min

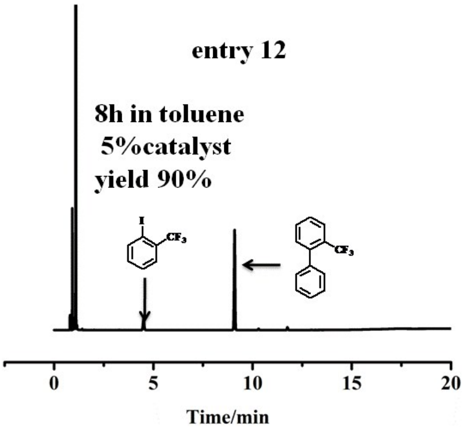
entry 12
8h in toluene
5%catalyst
yield 90%
0 5 10 15 20
Time/min

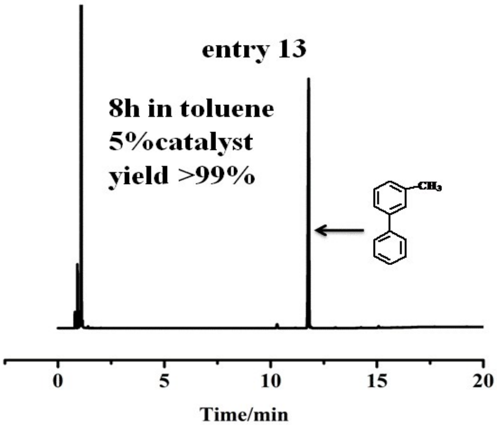
entry 13
8h in toluene
5%catalyst
yield >99%
0 5 10 15 20
Time/min

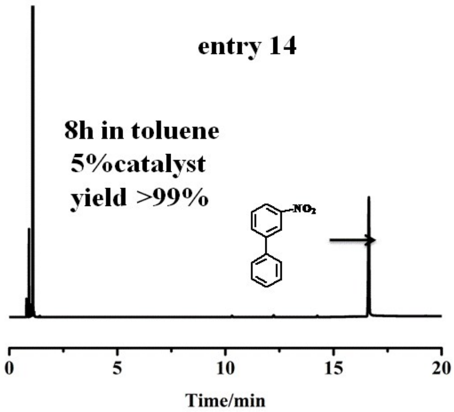
entry 14
8h in toluene
5%catalyst
yield >99%
0 5 10 15 20
Time/min

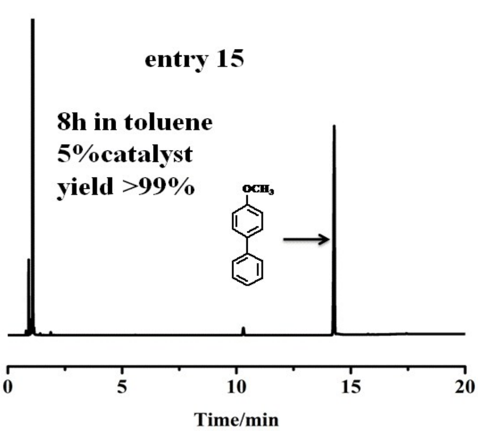
entry 15
8h in toluene
5%catalyst
yield >99%
0 5 10 15 20
Time/min

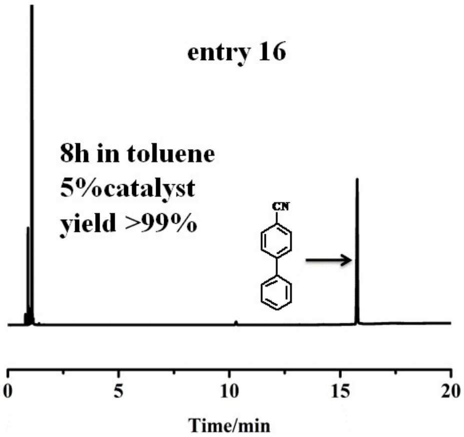
entry 16
8h in toluene
5%catalyst
yield >99%
0 5 10 15 20
Time/min

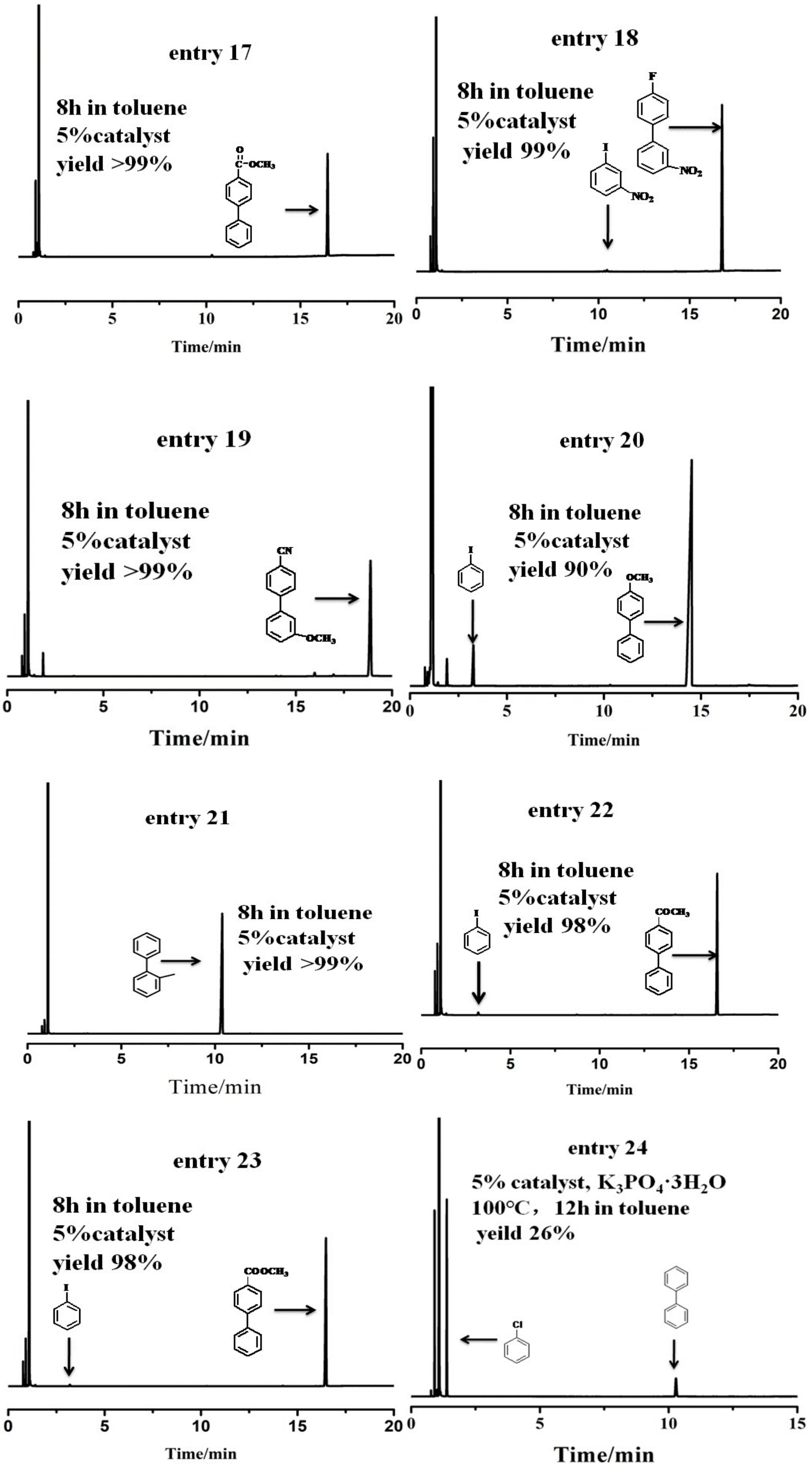

Fig. S7 GC results for the Suzuki cross-coupling reactions with various halogenobenzene and arylboronic acid substrates catalyzed by **Ni(II)-*α*-diimine-POP** in toluene (for Table 2).

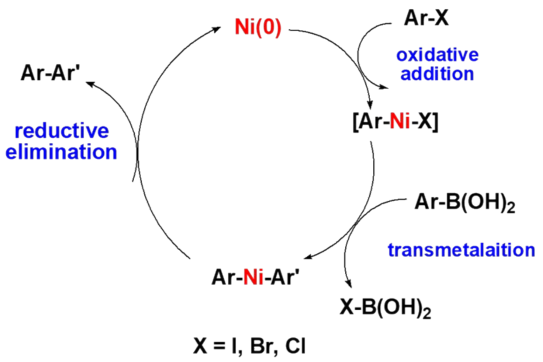

Fig. S8 Proposed mechanism for the **Ni(II)-α-diimine-POP** catalyzed Suzuki cross-coupling reaction.

Product characterization for Suzuki-Miyaura reaction[5]

(Compound i): ^{1}H NMR (400 MHz, $CDCl_3$, TMS) δ 7.60 (d, *J* = 8.0 Hz, 4H), 7.44 (t, *J* = 6.8 Hz, 4H), 7.35 (t, *J* = 7.2 Hz, 2H). ESI-MS m/z $[M+H]^+$= 177.0898 (cacld177.0910).

(Compound ii): ^{1}H NMR (400 MHz, $CDCl_3$, TMS) δ 8.31 (d, *J* = 8.8 Hz, 2H), 7.74 (d, *J* = 9.2 Hz, 2H), 7.63 (d, *J* = 6.8 Hz, 2H), 7.50 (t, *J* = 7.2Hz, 2H), 7.46 (t, *J* = 7.2 Hz, 1H). ESI-MS m/z $[M+H]^+$= 200.0730 (cacld200.0706).

(Compound iii): ^{1}H NMR (400 MHz, $CDCl_3$, TMS) δ 8.04 (d, *J* = 8.4 Hz, 2H), 7.69 (d, *J* = 8.4 Hz, 2H), 7.63 (d, *J* = 7.2 Hz, 2H), 7.48 (t, *J* = 7.6 Hz, 2H), 7.41 (t, *J* = 7.2 Hz, 1H), 2.64 (s, 3H). ESI-MS m/z $[M+H]^+$= 197.0975 (cacld197.0961).

(Compound iv): ^{1}H NMR (400 MHz, $CDCl_3$, TMS) δ 7.54 (t, *J* = 8.0 Hz, 4H), 7.41 (t, *J* = 4.0 Hz, 2H), 7.31 (t, *J* = 7.6 Hz, 1H), 6.98 (d, *J* = 8.8 Hz, 2H), 3.85 (s, 3H). ESI-MS m/z $[M+H]^+$= 185.0938 (cacld185.0961).

(Compound v): ^{1}H NMR (400 MHz, $CDCl_3$, TMS) δ 8.11 (d, *J* = 8.4 Hz, 2H), 7.66 (d, *J* = 8.4 Hz, 2H), 7.63 (d, *J* = 6.8 Hz, 2H), 7.47 (t, *J* = 7.2 Hz, 2H), 7.40 (d, *J* = 7.2 Hz, 1H), 3.94 (s, 3H). ESI-MS m/z $[M+H]^+$= 213.0925 (cacld213.0910).

(Compound vi): ^{1}H NMR (400 MHz, $CDCl_3$, TMS) δ 7.77(d, *J* = 7.6 Hz, 1H), 7.65 (t, *J* = 9.2 Hz, 1H), 7.55 (d, *J* = 6.8 Hz, 2H), 7.52 (t, *J* = 6.8 Hz, 3H), 7.43 (t, *J* = 4.8 Hz, 2H). ESI-MS m/z $[M+H]^+$= 180.0800 (cacld180.0808).

(Compound vii):^{1}H NMR (400 MHz, $CDCl_3$, TMS) δ 7.42(t, *J* = 8.0 Hz, 2H), 7.33 (m, 3H), 7.26 (m, 4H), 2.27 (s, 3H). ESI-MS m/z $[M+H]^+$= 169.1026 (cacld169.1012).

(Compound viii): ^{1}H NMR (400 MHz, $CDCl_3$) δ 8.46 (t, *J* = 2.0 Hz, 1H), 8.24-8.15 (m, 1H), 7.92 (d, *J* = 7.2 Hz, 1H), 7.63 (t, *J* = 6.8 Hz, 3H), 7.50 (t, *J* = 8.4 Hz, 2H), 7.43 (t, *J* = 7.6 Hz, 1H). ESI-MS m/z $[M+H]^+$= 200.0696 (cacld200.0706).

(Compound ix): ^{1}H NMR (400 MHz, $CDCl_3$) δ 7.58 (d, *J* = 7.6 Hz, 2H), 7.43 (d, *J* = 7.2 Hz, 2H), 7.39 (d, *J* = 8.8 Hz, 2H), 7.33 (t, *J* = 6.8 Hz, 2H), 7.16 (d, *J* = 7.2 Hz, 1H), 2.42 (s, 3H). ESI-MS m/z $[M+H]^+$= 169.1006 (cacld169.1012).

(Compound x): ^{1}H NMR (400 MHz, $CDCl_3$) δ 7.73 (d, *J* = 8.0 Hz, 2H), 7.68 (d, *J* = 8.4 Hz, 2H), 7.59 (d, *J* = 7.6 Hz,

2H), 7.49 (t,*J* = 7.2 Hz, 2H), 7.43 (t,*J* = 7.2 Hz, 1H). ESI-MS m/z $[M+H]^+$= 180.0833 (cacld180.0808).
(Compound xi):^{1}H NMR (400 MHz, $CDCl_3$) δ 7.53 (d, *J* = 7.2 Hz, 2H), 7.41 (t, *J* = 8.0 Hz, 2H), 7.35-7.29 (m, 3H), 7.03 (t, *J* = 6.4 Hz, 1H), 6.98 (d, *J* = 8.8 Hz, 1H), 3.81 (s, 3H). ESI-MS m/z $[M+H]^+$= 185.0944 (cacld185.0961).

(Compound xii): ^{1}H NMR (400 MHz, $CDCl_3$) δ 7.74 (d, *J* = 8.0 Hz, 1H), 7.55 (t, *J* = 7.6 Hz, 1H), 7.46 (t, *J* = 7.6 Hz, 1H), 7.41-7.38 (m, 3H), 7.33 (d, *J* = 4.8 Hz, 3H). ESI-MS m/z $[M+H]^+$= 223.0718 (cacld223.0729).

(Compound xiii): ^{1}H NMR (400 MHz, $CDCl_3$) δ 8.41(t, *J* = 4.0 Hz, 1H), 8.19 (m, 1H), 7.85 (m, *J* = 8.0 Hz, 1H), 7.61 (d, *J* = 4.0 Hz, 1H), 7.59 (m, 2H), 7.19 (m, 2H). ESI-MS m/z $[M+ H]^+$= 218.2065 (cacld218.2032).

(Compound xiv): ^{1}H NMR (400 MHz, $CDCl_3$) δ 7.73 (d, *J* = 12.0 Hz, 2H), 7.68 (d, *J* = 8.0 Hz, 2H), 7.39 (t, 1H), 7.18 (d, *J* = 12.0 Hz, 1H), 7.10 (t, *J* = 4.0 Hz, 1H), 6.97 (m, 1H), 3.87 (s, 3H). ESI-MS m/z $[M+H]^+$= 210.2500 (cacld210.2506).

References

[1] CrysAlisPro, Agilent Technologies, Version 1.171.36.32 (release 02-08-2013 CrysAlis171.NET) (compiled Aug 2 2013,16:46:58).

[2] SMART Version 5.625 and SAINT+ Version 6.02a. Bruker Analytical X-ray Systems, Inc., Madison, Wisconsin, USA, 1998.

[3] Sheldrick, G. M. SHELXTL Version 5.1; Bruker Analytical X-ray Systems, Inc., Madison, Wisconsin, USA, 1997.

[4] Spek, A. L. PLATON, A Multipurpose Crystallographic Tool. University of Utrecht, Utrecht, The Netherlands, 1998.

[5] (a) G.-Y. Liu, F.-W. Han, C.-X. Liu, H.-L. Wu, Y.-F. Zeng, R.-J. Zhu, X. Yu, S. Rao, G.-P. Huang, J.-H. Wang, *Organometallics.* 2019, **38**, 1459−1467. (b) T. E. Barder, S. D. Walker, J. R. Martinelli, S. L. Buchwald, *J. Am. Chem. Soc*. 2005, **127**, 4685-4696.
